# A Case of Iatrogenic Splenic Injury Following Routine Colonoscopy With Possible Influence of Unique Anatomy Due to Severe Scoliosis

**DOI:** 10.7759/cureus.14352

**Published:** 2021-04-07

**Authors:** Jacob B Wright, Sanjiv Gray, Dustin Huynh

**Affiliations:** 1 Surgical Critical Care, University of Central Florida College of Medicine, Orlando, USA; 2 Surgery, University of Central Florida College of Medicine, Orlando, USA

**Keywords:** splenic trauma, ­trauma, colonoscopy complications, splenic injury, splenic laceration, scoliosis, iatrogenic complications

## Abstract

Iatrogenic splenic injury is a rare complication of all abdominal surgeries. Despite the procedure’s overall safety, colonoscopy is now the procedure most frequently associated with iatrogenic splenic injury. A 58-year-old male with a past medical history of hypertension, lung cancer in remission, colon polyps, and severe scoliosis presented for grade three splenic laceration two days following a routine colonoscopy. He had no recent history of injury or other inciting events that could have led to traumatic injury. Non-operative management included splenic artery embolization and transfusion of one unit of packed red blood cells, after which he improved in the hospital and was discharged home in stable condition. This case postulates the possible influence of his severe scoliosis, and thus altered abdominal viscera anatomy, on his iatrogenic splenic injury, as well as the potential importance of investigating scoliosis as a risk factor for difficult colonoscopy or even iatrogenic splenic injury during colonoscopy.

## Introduction

Although the spleen is not a vital organ, it holds great clinical significance because injury to the spleen can be potentially life-threatening, as a ruptured spleen can cause extensive hemorrhage into the peritoneal cavity due to the densely vascular nature of the splenic parenchyma. Rupture of the spleen is characterized as a break to the splenic capsule and some degree of disruption to the parenchyma beneath. Trauma is the most common cause of splenic rupture, but non-traumatic causes have been reported, with mortality near 12% [1]. Causes of non-traumatic splenic rupture include neoplasm, infection, inflammatory disease, medication/treatment, mechanical issues, and idiopathic.

In writing this report, no literature was identified where altered anatomy due to scoliosis was documented or hypothesized as an increased risk for iatrogenic splenic injury following colonoscopy, or difficult colonoscopy. This case presents a likely iatrogenic splenic injury following esophagogastroduodenoscopy (EGD)/colonoscopy in a patient with severe scoliosis, and thus hypothesizes that altered anatomy posed an increased risk for the injuries obtained.

## Case presentation

A 58-year-old male patient with a past medical history of hypertension, lung cancer currently in remission (chemotherapy, radiation, and lobectomy), emphysema, scoliosis, and colon polyps presented to the emergency department as a transfer trauma alert from a nearby community hospital. He initially came to the hospital for persistent and worsening left upper quadrant (LUQ) abdominal pain, left shoulder pain, and decreased appetite for two days, which began immediately following routine colonoscopy for his history of polyps. His pain was worse with movement and minimally improved with rest. No polypectomy was done at this most recent colonoscopy. He denied any recent injuries or events such as motor vehicle accidents that could suggest traumatic injury, but did report that he had two episodes of near falls at home, where he got extremely lightheaded when bending down to dry himself after showering. He further denied any recent nausea/vomiting/diarrhea, bloody stools, chest pain, palpitations, prior abdominal surgeries, or use of aspirin or other anticoagulant medications.

**Figure 1:**
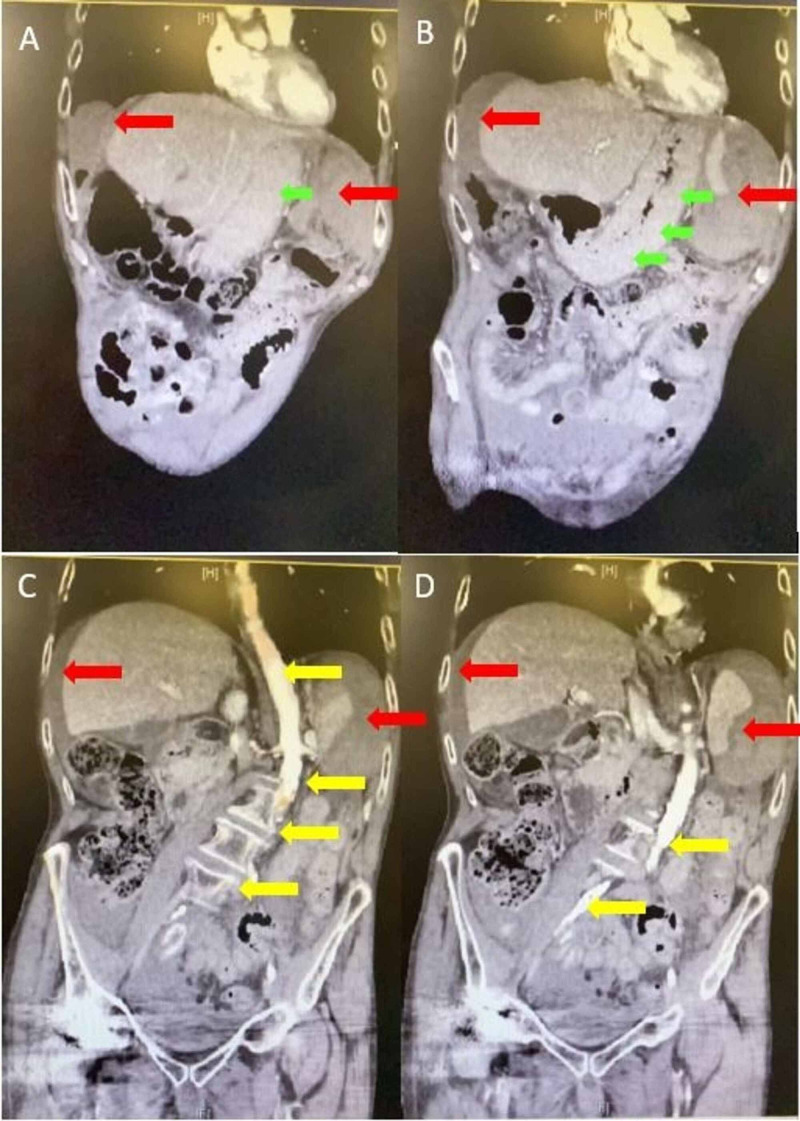
Series of four coronal images from abdominal CT. Splenic injury with hemoperitoneum (A-D, red arrows), scoliosis/angulated aorta (C and D, yellow arrows), stomach with severe wall thickening (A and B, green arrows). CT, computed tomography

**Figure 2:**
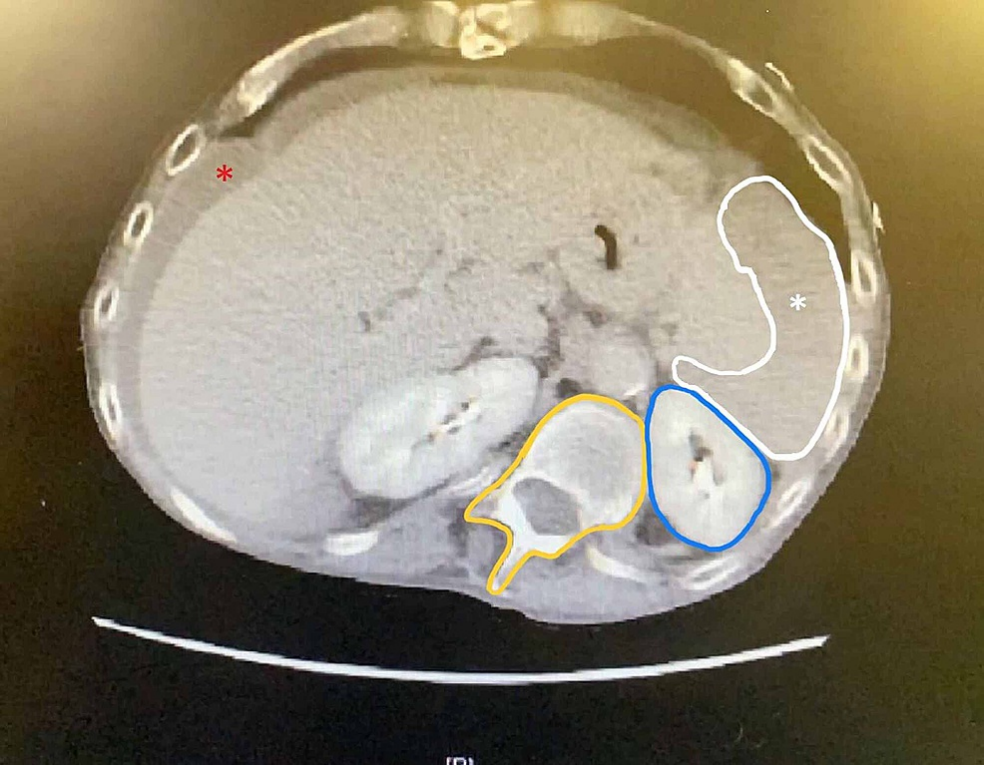
Axial view CT image. Extensive splenic hematoma (white asterisk and outline), hemoperitoneum (red asterisk), and scoliotic vertebrae (yellow outline) abutting the left kidney (blue outline) and injured spleen. CT, computed tomography

**Figure 3:**
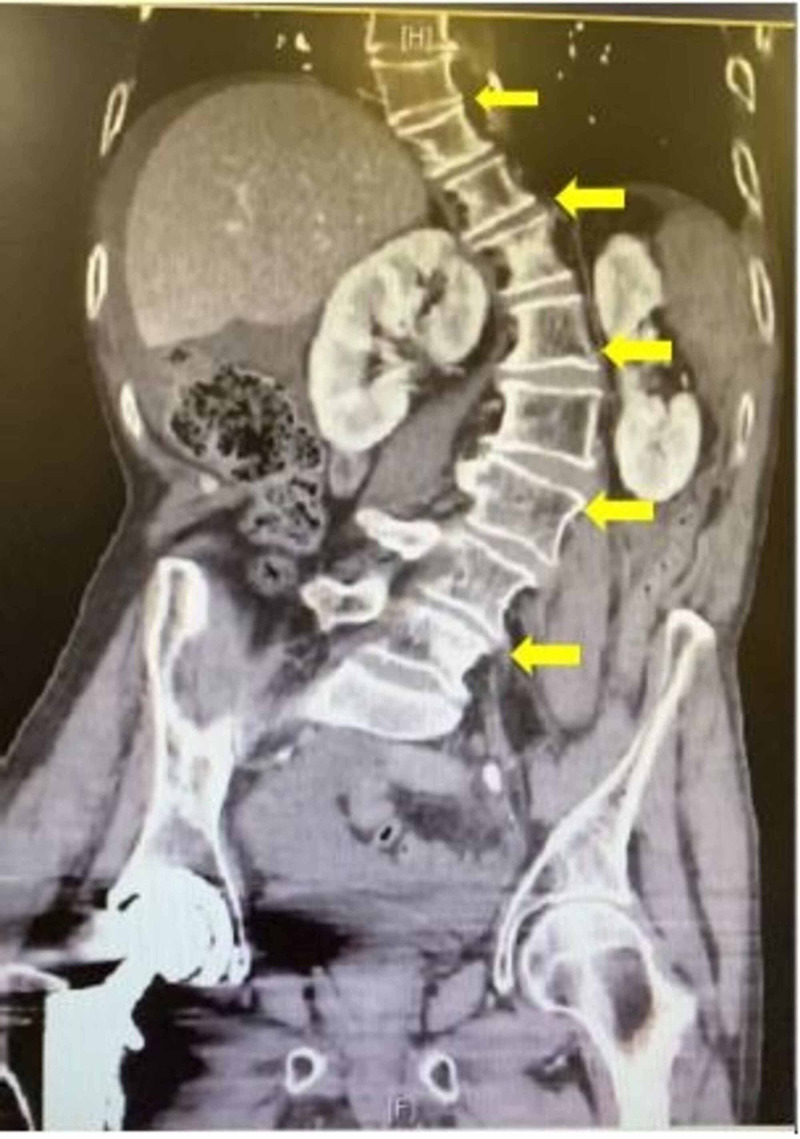
CT image depicting scoliotic vertebrae. CT, computed tomography

On initial assessment following transfer, he was lying in bed in no acute distress. There was no evidence of trauma or skin changes. His pain and tenderness to the LUQ were moderate but unchanged from when he left the previous facility. Vital signs included a temperature of 98.2 °F (36.78 °C), blood pressure 115/75 mmHg, pulse 99 beats/minute, respiratory rate of 20 breaths/minute, and oxygen saturation of 98% on 2 L oxygen delivered by nasal cannula. Complete blood count revealed hemoglobin of 6.5 gm/dL, prothrombin time was 13 seconds, partial thromboplastin time was 29 seconds, and international normalized ratio was 1.2. Computed tomography (CT) scans were obtained at the community facility. Initial radiologist documentation reported extensive perisplenic hematoma, a wedge-shaped region of low density along the lower pole of the spleen representing possible laceration, small amount of pooling of extravasated contrast material along the lateral lower pole aspect of the spleen on delayed phase imaging consistent with active extravasation, a large amount of hemoperitoneum but no free air, significant hemoperitoneum in the pelvis, and severe levoconvex changes and rotary scoliotic deformity in the proximal lumbar spine [Figures 1, 2, 3]. Overall impression directly from the radiologist’s documentation: “Large amount of hemoperitoneum with suspected grade three laceration along the lower pole aspect of the spleen with evidence of adjacent active extravasation.”

CT images from the initial facility were reviewed by the trauma surgeon, which prompted immediate in-person consultation with interventional radiology to discuss embolization versus laparotomy. The interventional radiologist agreed to perform endovascular embolization, and the patient was admitted to the neuro/trauma intensive care unit for close monitoring on intravenous pain control and anxiolytics before the procedure as he was hemodynamically stable. Splenic artery angiogram demonstrated laceration in the parenchyma but no evidence of active extravasation or blush. There was an additional accessory gastric artery arising from the splenic trunk, as well as what appeared to be an additional diaphragmatic/mesenteric branch. As there was active extravasation on CT with a large hemoperitoneum, proximal splenic artery embolization was performed with no complications. It was noted in the operative report that the embolization occurred distal to the additional/accessory branch vasculature. Postoperative hemoglobin of 6.5 gm/dL prompted transfusion of one unit of packed red blood cells, which improved his hemoglobin to 8.2 gm/dL. He was not transfused any blood products prior to the procedure and did not receive any subsequent transfusions. The patient continued to recover well in the hospital and was discharged home in stable condition after three days.

## Discussion

Colonoscopy is the gold standard, routine screening, and therapeutic procedure for lower intestinal disease and malignancy, and is regarded as generally safe. While splenic injury is an uncommon complication of colonoscopy, its mortality rate of 5% [2] and seemingly increasing incidence of case reports have led to recent literature exploring potential causes, risk factors, and management practices. A review of the literature showed excess torque at the splenic flexure of the colon, and thus tension on the splenocolic ligament, to be a frequent mechanism of damage to the spleen [2-12]; however, the authors were unable to identify any previous report of scoliosis and altered anatomy as a contributing risk factor for increased tension on the splenocolic ligament or iatrogenic splenic injury during colonoscopy. Numerous factors for predicting difficult colonoscopy have been reported [13-15], but scoliosis has not been noted as a predictive factor.

Splenic injuries are classified by CT findings, ranging from small subcapsular tear and less than 1 cm parenchymal laceration depth (grade one) to completely shattered spleen or injury to vascular or hilar structures that result in spleen devascularization (grade five). The American Association for the Surgery of Trauma provides grading for spleen injuries [16]. In our case, the criteria interpreted by the radiologist likely included subcapsular hematoma greater than 50% of the spleen surface area, and possibly parenchymal laceration greater than 3 cm depth [16].

Organ injury classification guidelines note that this grading system is useful for injury categorization, but should not solely be used to direct non-operative versus surgical intervention [16]. Treatment is also dependent on the etiology of rupture, as well as hemodynamic stability of the patient. Management options include non-operative management, selective or non-selective angioembolization, and splenorrhaphy or splenectomy. Practice has shifted to favor conservative management with hemodynamically stable patients compared to the rate of splenectomy in the past. Embolization is favorable in that splenic function is largely preserved, with evidence supporting conserved phagocytosis and spleen-dependent T-cell immunity [17]. Thus, there is no need for post-embolization vaccination, in contrast to splenectomy. It has additionally been shown that, overall, the use of splenic artery embolization significantly increases the rate of spleen salvage in patients managed non-operatively [18].

Iatrogenic injuries are an important albeit uncommon cause of splenic injury. Some major abdominal surgeries that pose risk for iatrogenic splenic injury include left hemicolectomy, open anti-reflux procedures, left nephrectomy, and procedures involving exposure/reconstruction of the proximal aorta and its branches [3]. A study with 975,825 patients conducted from 2006 to 2008 by Masoomi et al. found that the rate of splenic injury for those undergoing colorectal resection was 0.96% [19]. Surprisingly, however, the procedure most commonly associated with iatrogenic splenic injury according to more recent literature is a common, minimally invasive procedure: colonoscopy. Recent literature also recognizes that iatrogenic splenic injury has been under-reported and under-detected in the past [4,5,10]. As more reports of splenic injury due to colonoscopy are documented, prompt recognition of iatrogenic splenic injury is crucial as it carries an overall mortality rate of 5% [2]. Some patient-dependent risk factors for spleen injury during colonoscopy include splenomegaly, adhesions from prior surgery, neoplasm, anticoagulation, concurrent inflammatory processes such as diverticular disease, pancreatitis, inflammatory bowel disease, endometriosis, and infectious processes, including but not limited to malaria, typhoid fever, and Epstein-Barr virus mononucleosis. Some operator-dependent risk factors include polypectomy, supine position, lack of experience, biopsy, excess traction, direct injury, and multiple previous colonoscopies. Excess traction on the splenocolic ligaments, and risk factors that would contribute thereof, have been postulated as the most likely causes of iatrogenic splenic injury [5]. Along with the identified risk factors for iatrogenic splenic injury, risk factors for difficult colonoscopy identified in the literature include colon loops or angulation, diverticular disease, low or high body mass index, older age, surgical adhesions, and female sex [13-15].

CT images included in the case presentation depict severe scoliosis as indicated by the radiologist; however, a review of the patient’s medical record revealed that pre-operative CT report one month before the EGD/colonoscopy did not include any information on altered anatomy due to scoliosis or acute angulation of the colon at the splenic flexure. Only general osseous degenerative changes and diffuse thickening of the stomach were noted, both of which were stable from prior imaging. It has been documented that marked scoliosis can cause significant anatomical change related to the spleen, and even an incident of intrathoracic spleen superior to the left ventricle was noted by Pacheco et al. [20]. In the case of our patient, it is clear that the severity of his scoliosis led to anatomical changes of the abdominal viscera that were quite significant.

## Conclusions

The authors postulate the possibility that this patient’s scoliosis, and the resulting anatomical changes, led to increased tension on the splenocolic ligament, directly contributing to splenic injury during his colonoscopy. Furthermore, it is possible that due to the lack of reported anatomical anomalies shown on CT, the gastroenterologist was not aware of the patient’s severe scoliosis before initiating colonoscopy, and may have approached the splenic flexure with greater caution if he/she was aware. The authors acknowledge the importance of recognizing scoliosis as a possible risk factor for iatrogenic injury during colonoscopy. The authors additionally recognize that there is very little, if any, documentation of previous cases that reflect the events that occurred with this patient and that propose the influence of scoliosis and resulting altered anatomy on splenic injury risk during colonoscopy. Scoliosis, especially in severe cases, combined with other documented risk factors, should raise caution for the gastroenterologist performing colonoscopy, and more research is needed to elucidate the extent to which scoliosis is a risk factor for iatrogenic complications.
